# A Glial Variant of the Vesicular Monoamine Transporter Is Required To Store Histamine in the *Drosophila* Visual System

**DOI:** 10.1371/journal.pgen.1000245

**Published:** 2008-11-07

**Authors:** Rafael Romero-Calderón, Guido Uhlenbrock, Jolanta Borycz, Anne F. Simon, Anna Grygoruk, Susan K. Yee, Amy Shyer, Larry C. Ackerson, Nigel T. Maidment, Ian A. Meinertzhagen, Bernhard T. Hovemann, David E. Krantz

**Affiliations:** 1 Gonda (Goldschmied) Center for Neuroscience and Genetics Research, David Geffen School of Medicine at University of California Los Angeles, Los Angeles, California, United States of America; 2 Fakultät für Chemie und Biochemie, Ruhr-Universität Bochum, Bochum, Germany; 3 Life Sciences Centre, Dalhousie University, Halifax, Nova Scotia, Canada; 4 Hatos Center for Neuropharmacology, David Geffen School of Medicine at University of California Los Angeles, Los Angeles, California, United States of America; Dartmouth College, United States of America

## Abstract

Unlike other monoamine neurotransmitters, the mechanism by which the brain's histamine content is regulated remains unclear. In mammals, vesicular monoamine transporters (VMATs) are expressed exclusively in neurons and mediate the storage of histamine and other monoamines. We have studied the visual system of *Drosophila melanogaster* in which histamine is the primary neurotransmitter released from photoreceptor cells. We report here that a novel mRNA splice variant of *Drosophila* VMAT (DVMAT-B) is expressed not in neurons but rather in a small subset of glia in the lamina of the fly's optic lobe. Histamine contents are reduced by mutation of *dVMAT*, but can be partially restored by specifically expressing DVMAT-B in glia. Our results suggest a novel role for a monoamine transporter in glia that may be relevant to histamine homeostasis in other systems.

## Author Summary

Neurons, the cells in the brain responsible for carrying information, communicate with each other using a class of chemicals known as neurotransmitters. One family of neurotransmitters, the monoamines, includes dopamine, serotonin, and histamine, all of which play major physiological roles. However, unlike dopamine and serotonin, the regulation of the brain's histamine content is poorly understood. We are using the fruitfly *Drosophila melanogaster* to study the storage and release of histamine from brain cells. Both mammals and insects use a class of proteins called transporters to store amines, but, to date, amine transporters have been thought to be restricted to neurons. We have found that the support cells, or glia, that facilitate the function of neurons in the fly's visual system contain a new form of monoamine transporter. Despite its circumscribed distribution, this protein is required to maintain normal levels of histamine throughout the visual system. We speculate that other animals may use a similar strategy to regulate the function of this important neurotransmitter.

## Introduction

Histamine was first identified as a potential neuromodulator at the turn of the last century, and is now known to regulate multiple physiological processes in mammals as well as invertebrates [1]–[9]. For all other classical neurotransmitters, the transport proteins responsible for neurotransmitter storage and recycling play a critical role in regulating the amount of transmitter that is available for signaling at the synapse [10],[11]. Therefore, to understand the mechanisms by which histaminergic signaling is regulated, it will be critical to determine the transporters and transport mechanisms by which histamine and its metabolites are stored, released and recycled.

Both cell surface and vesicular transporters are required for neurotransmitter release and recycling. All classical neurotransmitters are synthesized in the cytoplasm and therefore must undergo transport into the lumen of secretory vesicles for regulated release. Vesicular neurotransmitter transporters for most known neurotransmitters have been identified and include the vesicular glutamate (VGLUT1, 2 and 3) [12], GABA/Inhibitory Amino Acid (VGAT/VIAAT) [13], acetylcholine (VAChT) and monoamine transporters (VMAT1 and 2) [14]. In mammals, histamine is transported into synaptic vesicles and secretory granules by the neuronal isoform of VMAT, VMAT2 [15]–[18].

After exocytotic release from the nerve terminal, neurotransmitters can be recycled via either direct or indirect routes, and each requires a distinct set of cell-surface transporters [19]. The plasma membrane transporters responsible for the specific, high affinity uptake of dopamine (DAT), serotonin (SERT), and noradrenalin (NET) are well-characterized and localize primarily to presynaptic nerve terminals [20]. Their localization is consistent with a role in directly recycling monoamines for immediate re-release, through re-uptake. In contrast, glutamate is primarily transported by the excitatory amino acid transporters (EAATS) into glia rather than the nerve terminal [21]–[23]; it is metabolized to glutamine in glia by the enzyme glutamine synthase [24]. Glutamine is exported from glia via efflux through system N transporters and then transported into glutamatergic neurons by system A [25],[26].

Since histamine is a monoamine neurotransmitter, its re-uptake might be expected to occur at presynaptic nerve terminals, as for other monoamines [9]. However, to date, a histamine transporter has not been identified in neurons. Rather, in mammals, astrocytes take on this role. For example, they take up radiolabeled histamine, possibly via non-specific organic cation transporters (OCTs), and express the enzymes responsible for histamine metabolism [27]–[32]. In mammals it is possible that histamine, unlike all other neurotransmitters, is not recycled, but is degraded, presumably in astrocytes. Alternatively, histamine, like glutamate, might be recycled via a relatively circuitous route that requires transport and metabolism in glia followed by re-export to neurons.

The *Drosophila* visual system is a useful system in which to study histamine release and recycling [9]. Histamine is the primary neurotransmitter released from insect and other arthropod photoreceptors [33]–[35], and many of the molecular elements required for histaminergic neurotransmission have been identified in the fly's visual system [33], [36]–[39]. As for mammals, histamine is synthesized in *Drosophila* by histidine decarboxylase, which localizes to the presynaptic site of histamine release, the photoreceptor terminal [34]. However, unlike mammals, it is unclear how histamine is transported into synaptic vesicles, since the *Drosophila* orthologue of VMAT is absent from fly photoreceptors [40]. Neurotransmitter release is both tonic and graded at photoreceptors [41] and in *Drosophila* occurs at a rate sufficiently high to require active mechanisms for recovery [9],[42]. Changes in the amount of histamine release and, perhaps more importantly, its removal from the synaptic cleft, are presumed to signal to interneurons and their ascending visual pathways [9]. However, it is still not known how histamine concentration in the synaptic cleft is controlled, nor is it clear how changes in synaptic histamine might affect the higher functions of the visual system.

Histamine is metabolized in *Drosophila* by the product of the gene *ebony* [43], which conjugates histamine to β-alanine to generate the metabolite β-alanyl-histamine, or carcinine [44],[45]. The gene product of *tan* mediates the hydrolysis of carcinine, and thereby the liberation of histamine [36]. Interestingly, Tan is localized to photoreceptors, the site of histamine synthesis, whereas *ebony* is expressed in the epithelial glia that surround the photoreceptor terminals [37],[45]. The reciprocal localization of Tan and Ebony, to neurons and glia respectively, implies that at least in *Drosophila*, histamine is recycled via a relatively complex pathway that involves uptake into glia [9]. Recent genetic experiments suggest that the gene *inebriated* (*ine*) might function as a carcinine transporter to allow metabolized histamine to be taken up by photoreceptor cells [46]. However, the transporters required for histamine uptake into glia and the mechanism by which carcinine is exported from glia are still not known. Moreover, with the possible exceptions of the OCTs and Inebriated, the transporters responsible for histamine uptake and homeostasis in both mammals and invertebrates likewise remain obscure.

To investigate the regulation of aminergic signaling in the fly, we have previously identified the *Drosophila* orthologue of the vesicular monoamine transporter (*dVMAT*) [40],[47]. The *dVMAT* gene expresses two splice variants (DVMAT-A and -B) that differ at their extreme C-termini [47]. This domain is required to traffic mammalian VMAT2 and VACHT to synaptic vesicles and other types of secretory vesicles [48]–[54]. As for mammalian VMATs, DVMAT-A is expressed in all aminergic neurons in the fly CNS [15]–[17],[40],[47],[55]. We show here that DVMAT-B is not expressed in neurons, but rather in a subset of glia that are adjacent to the retina and store histamine. Furthermore, the loss of DVMAT-B's function at this site reduces histamine storage. These data indicate that, unlike other VMATs, DVMAT-B has a functional role in glia rather than neurons, and that DVMAT-B helps to regulate histamine in the fly's visual system.

## Results

**Figure 1:**
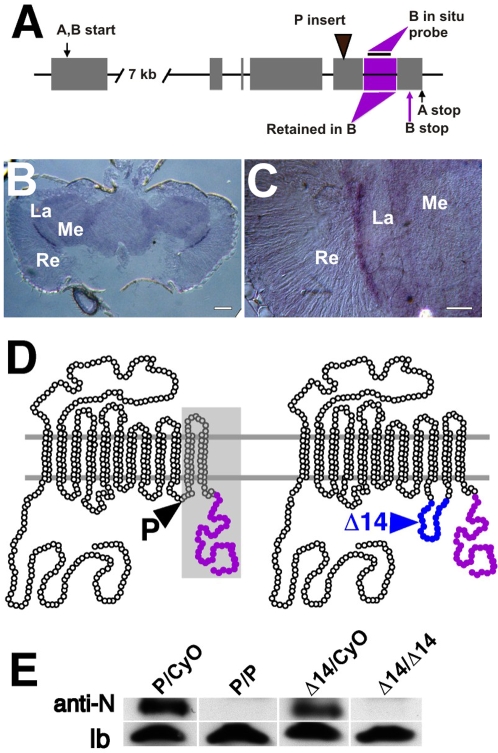
Mutant alleles of *dVMAT* reduce expression of DVMAT-A and -B. (A) *dVMAT-A* and *-B* share a common translational start site (indicated as “A, B start”) and a common N-terminus, but diverge at their C-termini. Coding exons in the *dVMAT* gene that are common to both *dVMAT*-*A* and-*B* are shown as gray boxes, introns as black lines. To generate alternative carboxy termini, the indicated genomic sequence (magenta box) is spliced out from *dVMAT-A* and retained in *dVMAT-B*. The *in situ* probe for *dVMAT-B* contains the first 260 nucleotides of this sequence. The P element insert in the *dVMAT* mutant allele *dVMAT* ^P1^ (black arrowhead) disrupts the coding sequence of both *dVMAT-A* and *-B*. (B,C) *In situ* hybridization of head sections shows transcription of the *dVMAT-B* gene in a layer between the retina and lamina (B). Magnified view of (B) shown in (C). (D) Cartoons showing the predicted topology of DVMAT with lumenal domains above and cytoplasmic domains below the parallel gray lines representing the vesicle membrane. Open circles indicate domains shared by DVMAT-A and -B. Filled, magenta circles indicate the C-terminal domain specific for DVMAT-B. The P element insertion site in the last exon of the *dVMAT* ^P1^ (marked with “P” and a black arrowhead) functionally deletes transmembrane domains 11 and 12 and the C-terminus (shaded gray). The imprecise excision allele *dVMAT* ^Δ14^ results in an insertion of 51 base pairs, and 17 amino acids in-frame with the original downstream codons (marked as “Δ14” with a blue arrowhead, with blue circles indicating the inserted residues). (E) Western blot using the N-terminus antibody directed against both DVMAT-A and -B splice variants shows an absence of DVMAT protein in *dVMAT* ^P1^ homozygotes (P/P), and dramatically reduced levels in *dVMAT* ^Δ14^ (Δ14/Δ14), compared with heterozygous controls (P/CyO and Δ14/CyO). The plasma membrane associated protein Late Bloomer (indicated as “lb”) was used as a loading control. Re, retina; La, lamina; Me, medulla. Bars: (B) 50 microns, (C) 20 microns.

The mRNA of *dVMAT* is alternatively processed to yield two variants [47]. In *dVMAT-B*, the segment indicated in Figure 1A is retained. In *dVMAT-A*, all of the splice sites are used and the indicated segment is removed as an intron. To determine the expression pattern of *dVMAT-B* mRNA in the adult brain, we performed *in situ* hybridization experiments. To generate a cDNA probe specific for *dVMAT-B*, we amplified the sequence retained in *dVMAT-B* mRNA and removed from *dVMAT-A* (Figure 1A, “B *in situ* probe”). Using this probe we observed a narrow band of labeling in the region just beneath the retina (Figure 1B and 1C). These data are consistent with a previous report showing that a probe common to both *dVMAT-A+B* labels a thin band below the retina in addition to aminergic neurons in the central brain and optic ganglia [56]. Based on this labeling pattern and proximity to the glial marker Neurexin IV, it was suggested that *dVMAT* might be expressed in the fenestrated glia [56].

To further elucidate the localization and function of DVMAT-B, we generated an antiserum to the N-terminus domain shared by DVMAT-A and -B, and two separate antibodies specific for the DVMAT-B C-terminus (see Methods for additional details). To demonstrate the specificity of our antibodies and to investigate the relationship of DVMAT-A and/or -B to histamine storage, we characterized a *dVMAT* mutant line. (A more complete phenotypic analysis of the *dVMAT* mutant will be reported elsewhere.) We have previously reported that *CG6119* encodes the 3′ portion of *dVMAT* [47], and have obtained a line (*l(2)SHO459*) containing a transposable P element in this predicted gene segment [57]. We used inverse PCR and DNA sequencing to confirm that the insertion site of *l(2)SHO459* is in the last coding exon of *dVMAT*. The insertion creates a functional deletion of the last two transmembrane domains of both DVMAT-A and -B (see Figure 1D, “P”). We therefore refer to *l(2)SHO459* as *dVMAT* ^P1^. Western blots of adult head homogenates were probed with the antibody directed to the N-terminus of DVMAT shared by the DVMAT-A and -B splice variants (anti-N). Little or no protein representing either DVMAT isoform remains in the homozygotes containing the P insertion as compared with controls (Figure 1E, compare lanes “P/CyO” and “P/P”). The absence of a detectable band in homogenates derived from the *dVMAT* ^P1^ mutant demonstrates that anti-N is specific for DVMAT.

To generate additional *dVMAT* alleles, we excised the P-element in *dVMAT* ^P1^. One allele, *dVMAT* ^Δ14^, removes most of the transposon including the *white* (*w*) eye marker gene but leaves behind 51 base pairs within the sixth and last coding exon of *dVMAT*. The insertion is in-frame and encodes an additional 17 amino acids (HDEITSSLLTLFHHELG). Immunoblots of *dVMAT* ^Δ14^ flies using anti-N show very small amounts of DVMAT (Figure 1E, “Δ14/Δ14”).

**Figure 2:**
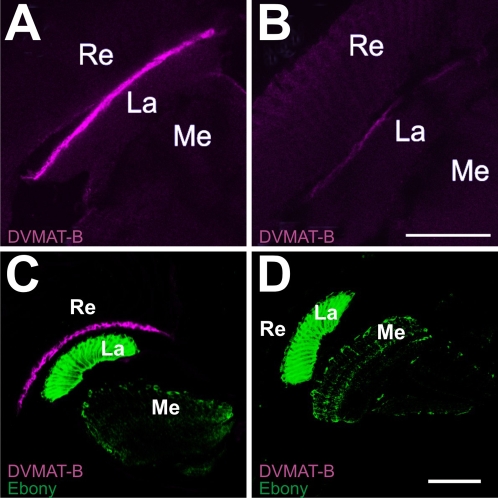
Antibodies raised against the C-terminus of DVMAT-B label the distal lamina. Confocal images of whole adult *w* and *dVMAT* ^Δ14^ mutant brains were labeled with the DVMAT-B antiserum anti-B_1_ (A,B). Labeling is visible in the distal lamina of the *w* control brain (A), but markedly reduced in the homozygous *dVMAT* ^Δ14^ mutant (B). Confocal images of *wt* and *dVMAT* ^P1^ homozygote head sections were labeled with anti-B_2_ (C,D) (magenta) and anti-Ebony (C,D) (green). In the *wt*, control sections, DVMAT-B labeling is visible in the distal lamina (C). In the *dVMAT* ^P1^ mutant, however, no DVMAT-B expression is detected (D). These results demonstrate the specificity of both anti-B_1_ and -B_2_. Re, retina; La, lamina; Me, medulla. Bars: 50 microns.

We next characterized the two C-terminus directed antibodies that we generated to specifically detect DVMAT-B. Since only the C-termini of DVMAT-A and -B differ, a peptide representing the last 21 amino acids of DVMAT-B was used to develop a polyclonal antibody specific to the B form (anti-B_1_, see Methods). Immunoblots using anti-B_1_ did not show a detectable band on Western blots using adult head homogenates that gave robust signals when probed with either anti-DVMAT-A or anti-N (not shown). Therefore, to validate the specificity of the antibody and to determine the expression pattern of the DVMAT-B splice variant, we performed immunolabeling experiments using whole adult brains. In contrast to our previously described antibody to DVMAT-A (see [40],[47]), aminergic neurons in the adult brain were not labeled with anti-B_1_. Rather, anti-B_1_ specifically labeled a relatively thin layer between the retina and the optic lobe (Figure 2A). To confirm the specificity of labeling using anti-B_1_, we repeated this experiment using the *dVMAT* ^Δ14^ flies. Labeling using anti-B_1_ was dramatically reduced in the *Δ14* mutants (Figure 2B), confirming the specificity of the anti-B_1_ antibody. Additional labeling experiments using anti-B_1_ indicate that DVMAT-B is not expressed elsewhere in the adult fly brain (data not shown). Despite the presence of *dVMAT-B* mRNA in the embryonic nervous system [47] we did not detect DVMAT-B protein in labeling experiments using anti-DVMAT-B in either whole embryos, the central nervous system of third-instar larvae (central brain plus optic lobes), or larval fillets that included Type II neuromuscular junctions, which are octopamine positive [58].

A previous study has shown that an mRNA *in situ* probe for *dVMAT-A+B* labels cells beneath the basement membrane that correspond in position to those of fenestrated glia [56]. Our *in situ* results and immunolabelings using anti-B_1_ confirm this pattern for both the *dVMAT-B* mRNA and the DVMAT-B protein. We were surprised by these results and the possibility that DVMAT-B might—as a result—localize to glia, since mammalian VMATs localize exclusively to neurons and neuroendocrine cells [15]–[17],[59]. We therefore performed additional experiments using a second antibody that was independently generated against the C-terminus of DVMAT-B (anti-B_2_.). We used anti-B_2_ to label cryosections, and co-labeled with an antibody to Ebony to help demonstrate the localization of the DVMAT-B label. Ebony labels a defined subset of glia in the lamina and medulla (the epithelial glia; [45]). Using anti-B_2_ we obtained results similar to those obtained in experiments using anti-B_1_; we observed a single band of labeling in *wt* flies that was absent in *dVMAT* ^P1^ flies (Figure 2C and 2D) between the retina and the lamina, and absence of labeling at other sites in the adult brain. These data confirm the specificity of anti-B_1_ and -B_2_ and indicate that DVMAT-B protein is expressed in a single relatively restricted region of the adult brain, consistent with our *in situ* probe of *dVMAT-B* mRNA.

**Figure 3:**
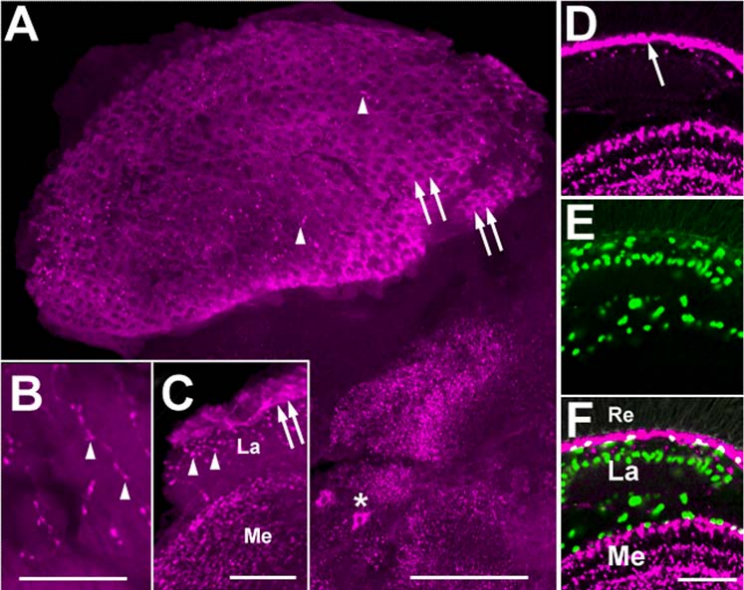
The antibody to DVMAT-A+B labels the optic neuropiles. (A) Projected confocal image of whole *w* adult brain labeled with the antibody against the N-terminus of DVMAT (anti-N) that recognizes both DVMAT-A and -B. The entire surface of the lamina is labeled with anti-N in a honeycomb pattern (white arrows). In the central brain, cell bodies (asterisk) and a large number of processes are labeled, consistent with the previously described expression of DVMAT-A. Additional punctate labeling in the lamina (B) (arrowheads) is likely to represent previously described serotonergic varicosities, more easily seen in a single optical section of the lamina (B) (arrowheads) and in a tangential section through the lamina (C) (La, see arrowheads) and distal medulla (C) (Me). Labeling of the lamina surface is also apparent (C) (arrows) in the tangential view of the lamina and distal medulla. (D–F) Cryostat sections of *w* adult brains labeled for Repo (green) and DVMAT-A+B (magenta). Repo label in glial cell nuclei of the optic lobe and central brain does not co-localize with the label for DVMAT-A+B, but the DVMAT-A+B label in the distal lamina appears to co-localize with glial cell nuclei (D) (arrow) (overlap shown in F). Bars: (A) 50 microns, (B,C) 25 microns, (D–F) 50 microns.

To determine the identity of the cells expressing DVMAT-B, we first performed additional experiments using the anti-N antibody directed against the N-terminus of DVMAT. Since the N-terminus of DVMAT is common to both DVMAT-A and B, labeling using anti-N showed the expression of both isoforms (DVMAT-A+B) and allowed the simultaneous visualization of both patterns of expression. In Figure 3A, labeling with anti-N in whole-mounts of the entire brain revealed a punctate pattern in the central brain and medulla as well as scattered cell bodies. This pattern was similar if not identical to the labeling in the adult brain we previously observed using an antibody specific for DVMAT-A [40]. Labeling with DVMAT-A also showed a punctate pattern in the lamina that represents projections from the LP_2_ cluster of serotonergic neurons [40]. Using anti-N to label DVMAT-A+B, we observed a similar punctate labeling pattern in the lamina (Figure 3A and 3B); however, unlike the pattern we saw using the antibody to DVMAT-A, the entire surface of the lamina was labeled by anti-N (Figure 3A, see also Figure 3C). These data are consistent with the expression pattern of DVMAT-B in the distal lamina that we observed using the anti-B antibodies.

The structure of the lamina has been analyzed in both the housefly and *Drosophila* [60]–[64]. The region expressing DVMAT-B in the distal lamina contains several layers of morphologically distinct glia. To determine whether the cells in the lamina that express DVMAT-B might indeed be glia, adult heads were fixed and sectioned using a cryostat and then double-labeled with the primary antibodies anti-N (Figure 3D) and a glia-specific marker, anti-Repo (Figure 3E). Labeling of cryostat sections with anti-N showed bands of punctate labeling in the medulla, consistent with previous labelings using the antibody to DVMAT-A [40]. Labeling with anti-N in the medulla revealed that there was a minimal overlap with glial cell nuclei, consistent with the exclusive localization of DVMAT-A to aminergic neurons in the central nervous system [40],[47]. In contrast, a band just beneath the retina labeled with anti-N (Figure 3D, arrow) appeared to overlap with Repo-labeled glial cell nuclei in the distal lamina (Figure 3F), supporting the possibility that DVMAT-B is indeed expressed in glia.

**Figure 4:**
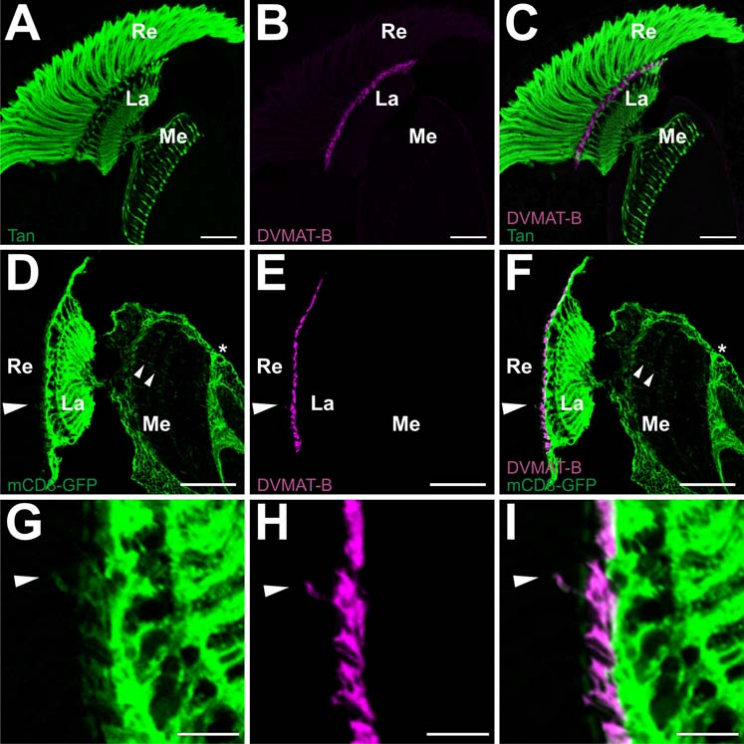
DVMAT-B is not detected in photoreceptor cells and co-localizes with a marker for *Drosophila* glia. A primary antibody to the protein Tan (A) (green) labels the photoreceptor cell bodies and their axons that extend into the lamina (La) and medulla (Me), seen in horizontal cryostat sections of the head. Co-labeling with anti-B_2_ (B) (magenta) shows no overlap with Tan in merged images (C). Glia were labeled using *repo-Gal4* to drive expression of the plasma membrane marker mCD8-GFP (D,G) (green). Some glial processes extend into the medulla (D,G) (small arrowheads). DVMAT-B was co-labeled using anti-B2 (E,H) (magenta). The merged images (F,I) show robust co-localization of DVMAT-B to profiles enclosed by glial cell membranes in the distal lamina, and additional, faint co-labeling of processes that extend distally into the retina (F,I) (large arrowheads). (G–I) Enlarged views of (D–F), to show the co-localization of the two signals. Bars: (A–F) 50 microns, (G–I) 10 microns.

To further examine the localization of DVMAT-B, we performed co-labeling experiments using the antibodies specific for DVMAT-B: anti-B_1_ and anti-B_2_. To establish the relationship of DVMAT-B labeling to photoreceptors, we first performed co-labelings using an antibody to the gene product of *tan*. Although originally identified as a mutation affecting pigmentation in the cuticle [43], Tan protein also localizes to photoreceptors, where it converts recycled carcinine to histamine [36],[37],[44]. Labeling with anti-B_2_ (red) and anti-Tan (green) revealed a mutually exclusive pattern of expression, indicating that DVMAT-B was not expressed in photoreceptor cells (Figure 4A–4C). Rather, it appeared to bracket the photoreceptor cell axons where they extended beneath the retina, in a position beneath the basement membrane. Co-labeling with anti-B_1_ and the photoreceptor specific antibody MAb24B10 [65],[66] confirmed this relationship (data not shown).

We next used the DVMAT-B specific antibodies to directly investigate the expression of DVMAT-B in glia. For these experiments, we used the line *repo-Gal4* to drive expression of mCD8-GFP and thereby label the plasma membrane of glial cells (Figure 4D–4I). This stratagem labeled glial cell membranes throughout the optic lobe and central brain (Figure 4D and 4G). Using the anti-B_2_ antibody we observed partial co-localization of DVMAT-B to the profiles enclosed by these glial cell membranes in the distal lamina (Figure 4F and 4I). As in the medulla, the glial processes in the distal lamina that extended toward the retina were less intensely labeled than other more proximal membranes (Figure 4D–4F), as clearly seen at high magnification (Figure 4G–4I). Nonetheless, these data strongly suggest that DVMAT-B is expressed in a subset of glial membranes abutting the retina. In addition, the localization and morphology of the DVMAT-B expressing cells suggests that they correspond to the fenestrated glia previously described in the housefly [62] (see Discussion).

**Figure 5:**
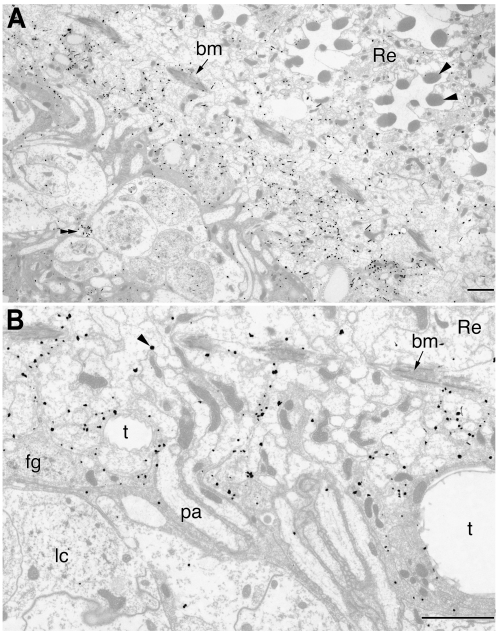
Immuno-EM shows that DVMAT in the distal lamina labels glia. (A) Electron micrograph of a tangential section through the proximal retina (Re) with seven rhabdomeres (arrowheads) visible in two complete cross-sections of ommatidia. The basement membrane, bm arrows in (A,B), separates the retina from the underlying lamina. Electron-dense gold particles lie just beneath the basement membrane. Beneath this band of labeling, an additional small profile quite distinct in appearance (double arrowhead) also expresses label, and may represent the profile of a serotonin-containing nerve terminal. (B) Higher magnification views of the labeled fenestrated glia and photoreceptor axons in the distal lamina. Gold particles (arrowhead) overlie the fenestrated glia. bm, basement membrane; pa, photoreceptor axons; t, tracheae; lc, glial nuclei. Bars: (A) 5 microns, (B) 2 microns.

Even though it has been suggested that *dVMAT* mRNA is expressed in the fenestrated glia [56], the localization of a vesicular monoamine transporter to glial cells had not been conclusively demonstrated. Both because of the heterodox nature of our observation and the difficulty inherent in interpreting co-localization from light micrographs, we performed additional immunolabelings at higher resolution, using electron microscopy (EM). For these experiments we used the anti-N antibody to visualize DVMAT-A+B; anti-N but not anti-B_1_ gave a good EM signal using the pre-embedding method. A high concentration of silver-enhanced gold particles was readily detected in the lamina, proximal to the basement membrane (Figure 5A), with some additional labeling seen in the retina itself. In addition, small profiles in the lamina cortex were also labeled. These may be profiles of serotonin-containing nerve terminals that express DVMAT-A [40], and are consistent with the punctate lamina labeling seen in light micrographs with anti-N (see Figure 3). The labeled glia were penetrated by ommatidial bundles of photoreceptor axons and also had extensive convolutions of their proximal cell surface (Figure 5B), consistent with the morphology of the fenestrated glia [62]. The convoluted morphology of the glia membranes made it difficult to determine the precise subcellular localization of DVMAT in material prepared by the pre-embedding method.

Previous electron microscopic analyses in the housefly have shown that two layers of glia occupy the region immediately beneath the basement membrane in the lamina. These include not only the fenestrated glia immediately abutting the basement membrane but also the pseudocartridge glia, which are proximal, i.e. closer to the central brain, relative to the fenestrated glia [61],[62]. Both are distal to the somata of the laminar cortex and, with the exception of the photoreceptor axons that penetrate these glia, neither the cell bodies nor processes of neurons occupy the region of the distal lamina that is labeled by DVMAT-B. Thus, the immuno-EM data support the conclusion that DVMAT-B is expressed in glial cells. Furthermore, DVMAT labeling is close to the basement membrane (Figure 5A and 5B), and additional label is seen in the proximal retina, consistent with our light micrographs (Figures 3 and 4). We therefore conclude that DVMAT-B expressing cells represent the fenestrated glia (see Discussion).

Previous immunohistochemical studies have shown that a similar region in the distal lamina just beneath the basement membrane is labeled with an antibody to histamine [44]. This location suggests that histamine could be contained in the fenestrated glia, but this interesting possibility has never been addressed. Furthermore, the fact that DVMAT-B localizes to cells in this region suggests that it might play a hitherto unacknowledged role in the glial storage of histamine in the *Drosophila* visual system. Importantly, the 12 transmembrane “backbone” required for transmitter transport is equivalent in DVMAT-A and -B, and we have shown that the DVMAT backbone common to DVMAT-A and -B recognizes histamine [47].

**Figure 6:**
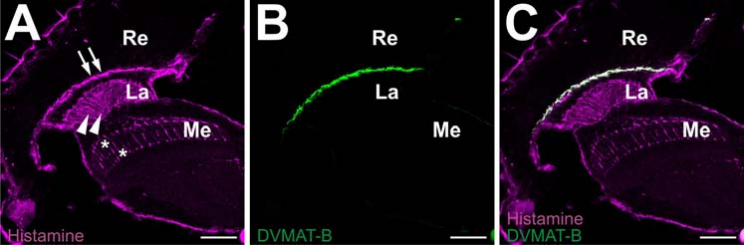
Co-labeling for DVMAT-B and histamine. (A) A primary antibody to histamine (magenta) labels photoreceptor cell terminals in the medulla (asterisks), an area in the proximal lamina (arrowheads) that contains the epithelial glia and axons of the outer photoreceptor cells, and a band just beneath the retina (arrows). The retina is weakly labeled in this section. (B) Co-labeling with anti-B_2_ (green) shows co-localization (C) with histamine in the band beneath the retina. Bar: 40 microns.

To immunolabel histamine in the fly's visual system, we used a previously characterized antibody and fixation protocol [67]. Consistent with previous reports [35],[44], the histamine antibody immunolabeled the retina, nerve terminals in the medulla, and much of the proximal lamina (Figure 6A). In addition, a band immediately beneath the retina in the distal lamina (Figure 6A) was labeled strongly for histamine. Strikingly, double labeling using anti-B_2_ showed robust co-localization (Figure 6B and 6C), suggesting that the same glial cells that express DVMAT-B also store histamine.

**Figure 7:**
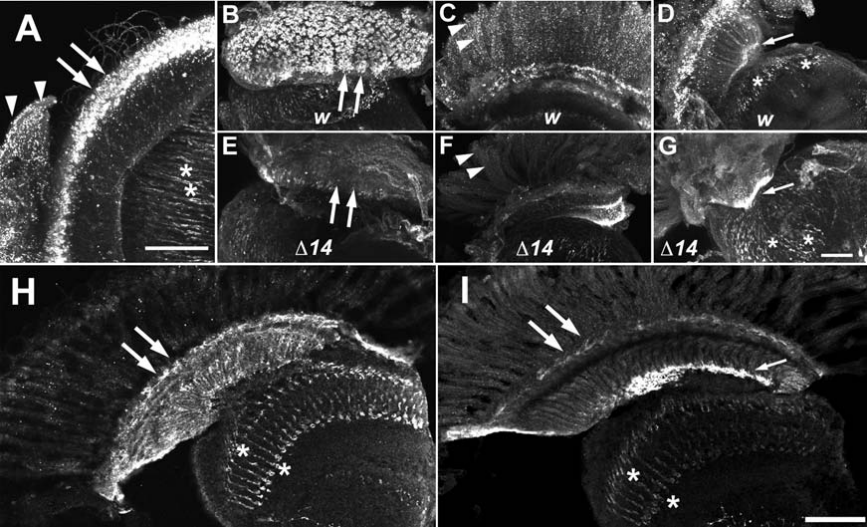
*dVMAT* mutants show decreased histamine labeling in subretinal glia. (A–G) Confocal projection images of whole-mount preparations of retina and optic lobes in control *w* (A–D) and mutant *dVMAT* ^Δ14^ homozygotes (E–G). Tangential views are shown in (A,C,D,F,G); the surface of the lamina is shown in (B) and (E). In *w* tissues, a primary antiserum to histamine shows labeling of the retina (A) (arrowheads), the distal lamina (arrows) and nerve terminals in the medulla (asterisks). *dVMAT* ^Δ14^ mutants (E–G) show reduced labeling at the surface of the lamina (E) (arrows) and retina (F) (arrowheads) relative to the lamina (B) and retina (C) in *w*. Labeling of nerve terminals in the medulla (D,G) (asterisks) and an area near the optic chiasm (D,G) (small arrow) in the *dVMAT* mutant (G) is less prominently reduced relative to the *w* control (D). (H) Cryostat sections of a *w* head labeled with the anti-histamine antibody also show labeling of the lamina, including a band just beneath the retina (H) (large arrows) as well as processes in the medulla (asterisks). Note that labeling of the retina is less evident in these cryostat sections compared with that in the confocal stacks shown in (A–G). (I) *dVMAT* ^Δ14^ shows a decrease in labeling in the distal lamina relative to *w—*compare (H) and (I) (large arrows). Labeling in the medulla (asterisks) is less reduced in *dVMAT* ^Δ14^ and is strong in the proximal lamina (small arrow). Bars: (A) 50 microns, (B–G) 25 microns, (H,I) 50 microns.

To explore this possibility further, and examine the possible functional role of DVMAT-B in histamine storage, we determined whether loss of *dVMAT* would decrease histamine labeling. To test this possibility, we used the same antibody against histamine to label brains from the *dVMAT* ^Δ14^ mutant and from *w* controls, and visualized the pattern of immunolabeling using confocal microscopy. In *w* optic lobes the antibody labeled the retina, distal lamina, and axon terminals in the medulla (Figure 7A, 7C, and 7D). Removal of the retina prior to labeling allowed visualization of the lamina surface and revealed a robust labeling pattern at the distal surface of the lamina in the *w* control (Figure 7B) that was dramatically reduced in the *dVMAT* ^Δ14^ homozygote (Figure 7E). This difference suggested that not only do the subretinal cells that express DVMAT-B store histamine, but also that DVMAT-B *function* in these cells is required for histamine storage.

Since neither DVMAT-A nor DVMAT-B are expressed in photoreceptor cells [40],[47], we were surprised to find that histamine labeling in photoreceptor cell bodies in the retina was also decreased in *dVMAT* ^Δ14^ relative to controls (compare Figure 7C and 7F, arrowheads). In contrast, *dVMAT* mutants showed no dramatic decrease in labeling for histamine in the proximal lamina near the chiasm, or in photoreceptor cell terminals in the medulla (compare Figure 7D and 7G). Thus, the presumptive role of DVMAT-B in neurotransmitter storage does not extend to all aspects of histamine homeostasis in the fly. Nonetheless, these data suggest that *dVMAT* may regulate histamine storage and homeostasis in the visual system in a more general fashion than might be expected based on its circumscribed pattern of expression.

Cryostat sections labeled for histamine also showed decreased labeling in the distal lamina and the retina in the *dVMAT* ^Δ14^ mutant (Figure 7I) relative to the *w* control (Figure 7H). The labeled sections also show that the residual labeling in the proximal lamina localizes to a region previously shown to contain another glia subset, the marginal glia [62],[68]. The pronounced labeling of this region suggests that histamine might be redistributed to an ectopic site in the *dVMAT* mutant.

**Figure 8:**
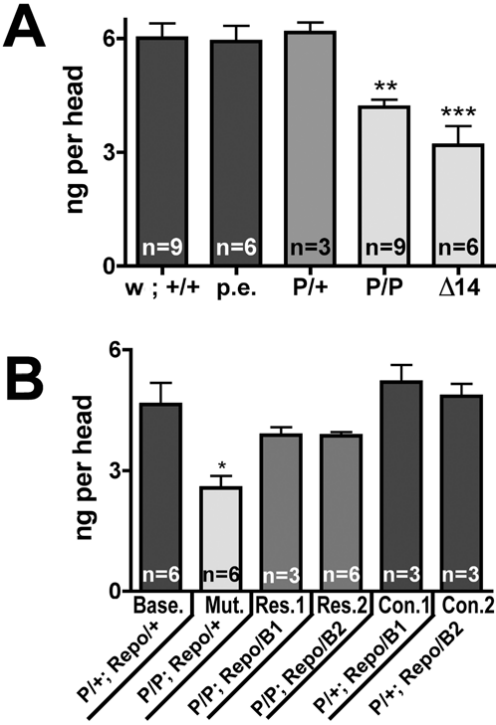
*dVMAT* mutant heads store less histamine than controls. (A) Histamine content is decreased by 30% in the homozygous *dVMAT* ^P1^ allele (P/P) and 47% in homozygotes of the *dVMAT* ^Δ14^ allele (Δ14) compared with the control line *w^1118^Cs*_10_ (w; +/+). Additional controls include a precise excision of the P in *dVMAT* ^P1^ (p.e.) and *dVMAT* ^P1^ heterozygotes (P/+); one-way ANOVA p<0.0001, with Bonferroni post-hoc test used to compare all data. Differences in head histamine from *w*;+/+ are indicated as **, p<0.01 and ***, p<0.001. Bars show the mean±SEM of independent trials measuring 3–4 heads/trial of randomly mixed sexes, with the number of trials (n) indicated in the bars. (B) Genetic rescue of *dVMAT-B*. All lines are in a genetic background containing the transgene *repo-Gal4*/+, and the genotype of the line used as a baseline control is *dVMAT* ^P1^/+; *repo-Gal4*/+ (indicated as “Base. P/+; Repo/+”). In this background, histamine content is reduced 45% by rendering the *dVMAT* ^P1^ allele homozygous (*dVMAT* ^P1^/*dVMAT* ^P1^; *repo-Gal4*/+, indicated as “Mut. P/P; Repo/+”. Rescue was performed using *repo-Gal4* with two separate *UAS-dVMAT-B* transgenes, *UAS-dVMAT-B1* and *-B2*, indicated as “Res.1 P/P; Repo/B1” and “Res.2 P/P; Repo/B2”, respectively. Additional controls include *dVMAT* ^P1^ heterozygotes with *repo-Gal4* and either *UAS-dVMAT-B1* or *-B2*, (indicated as “Con.1 P/+; Repo/B1”, and “Con.2 P/+; Repo/B2”). One-way ANOVA (p<0.0006, with Dunnett's multiple comparison tests) shows that “Mut.” head histamine content differs from all other lines (p<0.05: *). Bars show mean±SEM of independent trials measuring 4 heads, of randomly mixed sexes, with the number of trials indicated in the bars.

To quantify the contribution of DVMAT to histamine storage more accurately, we used high performance liquid chromatography (HPLC) to measure the total histamine content in *dVMAT* mutant heads (Figure 8A). As controls, we used: 1) *w^1118^Cs*_10_ (*w*; +/+), the genetic background into which *dVMAT* ^P1^ had been out-crossed; 2) a precise excision of the P element in *l(2)SHO459* (p.e.); and 3) *dVMAT* ^P1^ heterozygotes (P/+). The histamine content in heads derived from the three control lines—*w^1118^Cs*_10,_ p.e., and P/+—was 6±0.4, 5.9±0.4 and 6.1±0.3 and ng/head respectively. In contrast, the *dVMAT^P1^* (P/P) homozygotes contained 4.2±0.2 ng/head, a 30% reduction relative to the controls (Bonferroni post-test, p<0.01). The homozygous imprecise excision (*dVMAT* ^Δ14^, “Δ14” in Figure 8A), contained 3.2±0.5 ng/head, a 47% reduction relative to controls (Bonferroni post-test, p<0.001). These data indicate that *dVMAT* plays an unexpectedly important role in regulating the histamine content of the head, and together with the results from our histamine labelings they indicate that this role is exerted on the visual system by means of the fenestrated glia.

Finally, to address the role of DVMAT-B in the glial storage of histamine more specifically, we performed genetic rescue experiments (Figure 8B). To rescue the function of DVMAT-B in glia, we used the *repo-Gal4* driver to drive expression of the *UAS-dVMAT-B* transgene. We compared the histamine concentrations of heads from homozygous mutant flies containing *repo-Gal4* alone versus *repo-Gal4*+*UAS-dVMAT-B*. We note that *repo-Gal4* alone decreased histamine levels (compare Figure 8A and 8B). Therefore, all flies were tested in a *repo-Gal4* background, and our control for baseline histamine levels was *dVMAT* ^P1^/+; *repo-Gal4*/+ (indicated as “Base.” with the genotype abbreviated as P/+; Repo/+). The *repo*∶*dVMAT-B*, genetically rescued flies (“Res.1” and “Res.2”), showed a significant increase (Dunnett's multiple comparison test, p<0.05) in histamine relative to those containing *repo-Gal4* alone (indicated as “Mut.” P/P, Repo/+). These data indicate that expression of DVMAT-B in glia partially rescues the loss of histamine from the visual system.

## Discussion

As for glutamate, but unlike other biogenic amines, histamine recycling in *Drosophila* requires metabolism in nearby glia. Hitherto, the glial recycling pathway has been thought to be restricted to the epithelial glia that surround sites of lamina histamine release at the photoreceptor terminals [36],[37],[44],[45]. We now find that DVMAT-B localizes to a separate subset of glia that lie at the interface between the retina and the lamina, and that loss of DVMAT-B reduces histamine storage in the visual system, thus implicating these cells in the overall regulation of histamine after its release from photoreceptors.

**Figure 9:**
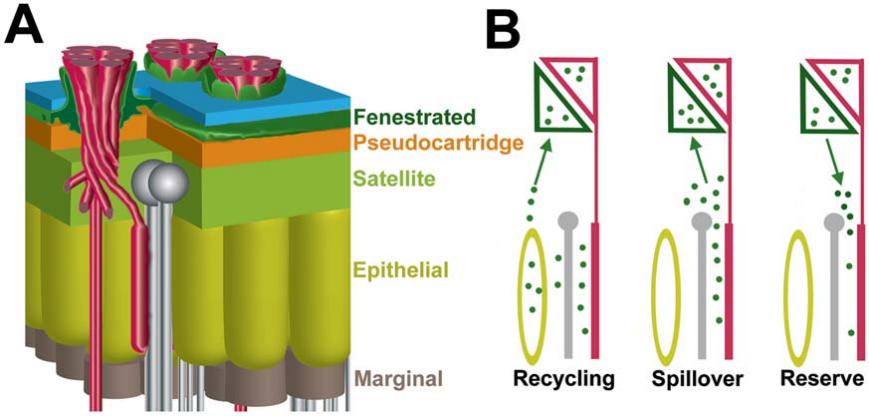
The location and possible function of the fenestrated glia. (A) Photoreceptor cells R1–R6 terminate in the lamina where they innervate lamina target neurons. Groups of six R1–R6 terminals and lamina target neurons are organized into cartridges, the component modules of the lamina. The diagram illustrates the relationship between the photoreceptor cell axons (red), the basement membrane (blue) separating the retina and lamina, and the lamina target neurons (silver). Identified glial subtypes in the fly's lamina include the fenestrated (dark green), pseudocartridge (orange), satellite (light green), epithelial (yellow), and marginal glia (brown). (B) Possible functions of DVMAT-B in the fenestrated glia include: recycling metabolized histamine back to the photoreceptors (“Recycling”); preventing spillover of histamine into nearby cartridges (“Spillover”); and releasing a reserve pool of histamine into the lamina to regulate its concentration during periods of heavy release (“Reserve”).

Ultrastructural and immunohistochemical studies in the fly have identified several distinct glial populations in the lamina. Although detailed ultrastructural accounts are available only in the housefly [61],[62], it is clear that *Drosophila* has similar populations of glia, and that genetic markers exist for most [68],[69]. The epithelial glia have been assigned an important role in histamine recycling [36],[44],[45], but the remaining glial subtypes have not been functionally characterized. The fenestrated glia lie closest to the retina and surround the photoreceptor axons as they enter the distal face of lamina (see Figure 9A). Processes from the fenestrated glia also extend through the basement membrane and into the retina. Our data strongly suggest that DVMAT-B localizes to the *Drosophila* equivalent of the fenestrated glia, consistent with the previously described location of the *dVMAT* transcript [56].

We find that DVMAT-B expression in glia is important for regulating the histamine content of the visual system. Mutation of *dVMAT* decreases histamine storage in the fly's head and expression of DVMAT-B in glia partially rescues this deficit. Importantly, immunolabeling for histamine in *dVMAT* mutants shows changes in both the fenestrated glia and photoreceptor cells, suggesting a more prominent role for DVMAT-B in histamine homeostasis than might be expected based on its limited pattern of expression.

We have shown previously that histamine and other monoamines are recognized by a DVMAT/VMAT2 chimera containing the domains common to DVMAT-A and -B [47]. These data support the idea that DVMAT-B could function as a histamine transporter in glia. It is also conceivable that DVMAT-B could recognize structurally related substrates such as the histamine metabolite carcinine. Although this remains speculative, it is useful to consider in the assessing the potential role of DVMAT-B in histamine homeostasis.

The location of DVMAT-B and the effects of the *dVMAT* mutant on histamine storage together suggest several potential functions for DVMAT-B. First, it is possible that DVMAT-B and the fenestrated glia play a role in histamine recycling. It is already known that histamine released from photoreceptor cell terminals in the lamina is metabolized to carcinine in the epithelial glia by Ebony [44],[45] and possibly transported into photoreceptor cells by Inebriated [46]. Carcinine is then converted back into histamine in the photoreceptors by Tan [36] to complete the recycling pathway. The shuttle pathway involving *ebony* and *tan* is very rapid [46]. Nonetheless, it is possible that carcinine produced by the epithelial glia could be stored in the fenestrated glia prior to its transport into the photoreceptor terminal, and that DVMAT-B allows the vesicular storage of carcinine and/or histamine in the fenestrated glia (Figure 9B, “Recycling” model). If DVMAT-B allows the fenestrated glia to function as an intermediate in the histamine-recycling pathway, it might be expected that the final step of the pathway, conversion of carcinine to histamine in the photoreceptors, would be blocked in *dVMAT* mutants. An elevation in carcinine is seen in *tan* mutants, and similarly, if DVMAT-B is required for recycling, mutation of *dVMAT* may elevate carcinine contents. In future experiments we will test this possibility using a previously developed assay [44] to analyze the effects of *ebony* and *tan* on histamine metabolism.

Second, it is possible that the fenestrated glia play a role in regulating the ‘spillover’ of any excess histamine that might diffuse away from its intended site of action, or otherwise accumulate ectopically after light-evoked release. Neurotransmitter transporters play an important role in regulating the amount of transmitter in the extracellular space that is available for signaling to postsynaptic receptors [70]–[74]. Inhibition of plasma membrane glutamate transporters, for example, increases glutamatergic signaling at extra-synaptic ionotropic receptors in the hippocampus [75] and cerebellum [76]–[78]. Glutamate spillover also regulates the activation of metabotropic receptors that localize to extrasynaptic sites on postsynaptic cells [79] and nearby neurons [80]. Similarly, GABA and dopamine transporters may regulate the amount of cross-talk that occurs between nearby synapses [81]–[83]. The position of the epithelial glia at the site of histamine release suggests that these rather than the fenestrated glia might control spillover into an adjacent synapse. In addition, the epithelial glia have recently been shown to express a histamine receptor, HclB (HisCl1) [84],[85] that could conceivably regulate histamine storage. However, it also remains possible that the fenestrated glia are involved in regulating possible spillover into more distal sites or over a longer time course. If this is indeed their primary role, loss of DVMAT-B activity would be predicted to cause a progressive, light-evoked decrease in spatial resolution through the excitation of neighboring cartridges, a possibility that optomotor turning responses at different light intensities [86] could reveal.

A third, recently described role for neurotransmitter transporters in glia is the storage and regulated release of transmitter. In addition to their well-established role in neurons, VGLUTs are also expressed in glia, and exocytotic glutamate release from astrocytes may regulate synaptic transmission [87]–[89]. Glial transporters also regulate extracellular levels of neurotransmitter through non-exocytotic mechanisms [90],[91]. In mammals, variations in the electrochemical potential across the plasma membrane of non-neuronal cells can promote GABA efflux through the GABA transporter [90]. A related transporter expressed in glia, xCT, uses an exchange mechanism to regulate glutamate levels at the *Drosophila* neuromuscular junction [91]. These studies highlight the emerging appreciation for glia as important sites of neurotransmitter release.

To assess the possibility that the function of the fenestrated glia may lie in this third role, to store and release histamine, it is useful to consider the unusual electrophysiological properties of the photoreceptor synapse. Since histamine activates a hyperpolarizing chloride channel [33], decreased histaminergic signaling depolarizes the target neurons in the lamina [9],[92]. Therefore, unlike most other synapses, the continuous presence of neurotransmitter in the synaptic cleft generates the resting state of the postsynaptic neuron, and decreases in cleft transmitter concentration depolarize the target neurons [9]. Thus, the constant presence of histamine in the synaptic cleft is required to maintain the postsynaptic target neurons in their normal state. We speculate that the fenestrated glia may provide a reserve pool of histamine for signaling in the lamina (Figure 9B, “Reserve”), with DVMAT-B serving to store and/or release the reserve pool. We would expect such release to occur under conditions of low neuronal histamine release, as when neuronal stores have by some means been depleted. A possible phenotype of the *dVMAT* mutant would be a reduced sensitivity, or altered rate of adaptation to light, testable using electroretinograms that report on neurotransmission at the photoreceptor synapse [93], or more direct intracellular recordings [94]. If glial histamine release facilitates adaptation, the response of the mutant would be expected to differ from *wt* under conditions of varying stimulus intensity. Regardless of whether the fenestrated glia provide a substrate for histamine recycling, spillover or reserve, further work will be required to resolve the relationships of these cells to histamine recycling in the epithelial glia [36],[37],[44],[45].

For each of the models we describe, it is possible that DVMAT-B functions in a manner similar to other vesicular transporters and mediates the storage of histamine in intracellular vesicles, albeit in glial rather than neuronal vesicles. Histamine could conceivably be stored in vesicles similar to those found in mammalian glia that release glutamate [87]–[89]. Alternatively, it is possible that DVMAT-B does not function as a classical vesicular transporter. The C-terminus of DVMAT-A is similar to the trafficking domains of mammalian VMATs and VAChT, and as for other vesicular transporters, DVMAT-A is efficiently endocytosed *in vitro* [47],[48]. In contrast, DVMAT-B contains a novel C-terminal domain, and is poorly endocytosed *in vitro* [47]. Indeed, most DVMAT-B appears to remain on the plasma membrane when it is expressed in cultured S2 cells [47]. It is therefore possible that DVMAT-B primarily localizes to the cell surface of the fenestrated glia *in vivo* as it does in S2 cells *in vitro*. Given that the fenestrated glia have extremely thin and highly convoluted processes, distinguishing between these possibilities will require additional, quantitative EM studies beyond those we report here.

If DVMAT-B localizes to plasma membrane *in vivo* as it does *in vitro*, its mechanism of transport would differ from other known vesicular transporters, all of which use a proton gradient to drive active transport. During periods of sustained neuronal activity, the extracellular milieu can acidify and the glial cytoplasm alkalinize [95]–[97]. The resultant, inwardly directed pH gradient could conceivably cause a vesicular transporter at the plasma membrane to transport neurotransmitter out of the cytoplasm and into the extracellular space. However, substrate exchange rather than active transport could also allow the export of histamine by DVMAT-B. Indeed, it is tempting to speculate that histamine stored in the fenestrated glia could be exchanged for extracellular carcinine. This idea is particularly attractive if DVMAT-B and the fenestrated glia serve to release histamine into the synaptic cleft as in the “Reserve” model. In this scenario, elevated levels of synaptic carcinine would directly activate the release of histamine into the cleft by the fenestrated glia. The exchange of histamine for carcinine would potentially serve both to maintain a baseline pool of histamine in the synapse while simultaneously sequestering carcinine for later recycling in photoreceptors.

Even though neurotransmitter metabolism in mammals differs from that in insects [43],[98], our results may bear on the transport mechanisms by which histamine homeostasis is maintained in mammals. Histamine uptake and metabolism in a variety of mammalian cells, including glia, is well established [27]–[32]. However, as in invertebrates, the plasma membrane transporters and putative recycling pathways for histamine both remain unclear. The unsuspected localization of DVMAT-B to glia and its role in regulating histamine levels in the fly raises the possibility that mammals may employ similar, novel mechanisms for the storage of histamine.

## Materials and Methods

### *In situ* Hybridization

For *in situ* hybridization, fly heads were mounted in Tissue-Tek O.C.T. compound (Microm, Walldorf, Germany) and were shock-frozen in liquid nitrogen. Sections (10 microns thick) were cut and fixed with 4% paraformaldehyde. After acetylation and prehybridization, subsequent hybridization with a digoxigenin-labeled *dVMAT-B* specific RNA probe (see Figure 1A) was performed overnight at 55°C. Specimens were blocked with normal goat serum in Tris-buffer saline / 0.1% Triton X-100 (TBT) and then treated with an alkaline phosphatase-coupled anti-digoxigenin antiserum (1∶1,000 dilution in TBT). NBT/BCIP color was developed overnight.

### *UAS-dVMAT-B* Lines

To facilitate the analysis of *dVMAT-B* transgenes, an hemagglutinin (HA) epitope tag was inserted at the identical site previously used for *dVMAT-A* [47]. The HA tag does not affect either expression or transport activity [47]. The HA-tagged construct representing the previously described coding sequence of *dVMAT-B* [47] was amplified using the polymerase chain reaction (PCR) and inserted into the expression construct *pEX-UAS* [99] to generate *UAS-dVMAT-B*. The sequence was verified at the UCLA Genotyping and Sequencing Core Facility and flies were transformed using standard methods [100] (Rainbow Transgenics, Newbury Park, CA). To test expression, 9 lines were crossed to the driver *daughterless-gal4* (*da-Gal4*) and homogenates from the progeny probed on Western blots as previously described using a primary antibody (HA.11, Covance Research Products, Denver, PA, USA) directed against the HA tag [40]. Two lines showing moderate levels of expression were chosen for genetic rescue experiments.

### Antibody Production

To generate an antiserum against the N-terminus of DVMAT shared by both the A and B splice variants (anti-N), a GST fusion protein containing the first 120 amino acids of DVMAT was generated. These residues represent the cytoplasmic N-terminus of DVMAT-A and -B, which precedes the first predicted transmembrane domain [47]. The relevant amplicon was generated using PCR and the primers AGGTGGAATTCAATCATCGACCGATG and ATACCCAAGCTTTCAGCGATTGGATCCCCGCCAG (including underlined EcoRI and HindIII sites respectively) and subcloned between the EcoRI and HindIII sites in the expression vector *pGEX-KG*, a gift of Greg Payne (UCLA), to generate a fusion with glutathione-S transferase (GST). The fusion protein was injected into rabbits (Cocalico Biologicals, Reamstown, PA, USA) and the antiserum affinity purified against the same fusion protein immobilized on nitrocellulose [47]. For Western blots and immunolabeling, anti-N was used at concentrations of 1/1000 and 1/250 respectively.

To generate an antiserum specific for DVMAT-B, an HPLC-purified peptide representing the last 21 amino acids of DVMAT-B (SVPDSDAEAGRTNEAYESERL, B1-peptide) was synthesized, conjugated to KLH and then injected into rats (Covance Research Products). The serum was affinity purified against the predicted carboxy terminus of DVMAT-B using a fusion protein GST-DVMAT-B containing the DVMAT-B specific carboxy terminal domain [47] immobilized on nitrocellulose. The affinity-purified antibody was used at dilutions of 1∶50 for immunohistochemistry.

A second antibody to DVMAT-B (anti-B_2_) was also raised in rats. The HPLC-purified peptide (H_2_N-GASVPDSDAEAGRTN+C-CONH_2,_ B2-peptide) was coupled to KLH and injected into rats (Eurogentec, Seraing, Belgium). The serum was affinity purified using B2-peptide conjugated to ACH-Sepharose. It was applied at a dilution of 1∶100 for immunohistochemistry.

### *dVMAT* Mutants

A fly line containing a P element insertion into the gene *CG6119* (the 3′ end of *dVMAT*) [47] has been previously isolated as an anonymous lethal gene on the second chromosome (*l(2)SHO459*) [57]. The line was a generous gift of Dr. S.X. Hou (NIH) and is maintained by the Indiana Stock Center (Bloomington, IL, USA) as a lethal mutation balanced over *CyO*. We designate this allele *dVMAT* ^P1^. To generate imprecise excisions from *dVMAT* ^P1^, the parent line was mated to a source of transposase (*Δ2-3*) and transposition allowed to occur in gametes of the F1 generation using standard genetic techniques. PCR amplification using a series of primers in the *dVMAT* gene was used to determine the approximate size of any deletions that occurred. PCR products from lines of interest were obtained from homozygous flies and sequenced (UCLA Genotyping and Sequencing Core). For the *Δ14* line, a 51 base-pair sequence from the P element was retained. This allele is designated as *dVMAT* ^Δ14^. Since *dVMAT* ^Δ14^ is phenotypically *white*, visual pigments that can impair immunolabeling experiments are absent. Another line showing a precise excision (*Δ10*) was used as a control and is indicated in the text as “p.e.”. Additional controls include Canton-S, and *w^1118^* outcrossed 10 times to *Canton-S (w^1118^Cs*_10_, indicated in the text as *w*; +/+) [101]. *dVMAT* ^P1^ was outcrossed 5 times to *w^1118^Cs*_10_ prior to genetic rescue experiments.

### Immunofluorescent Labeling

To immunolabel histamine in whole mounts, adult heads were manually dissected and fixed on ice in 4% 1-ethyl-3-(3-dimethylamino-propyl)carbodiimide (EDAC), 0.1 M Na_2_HPO_4_/NaH_2_PO_4_ buffer, pH 7.4 for 2.5 hours. For immunolabeling experiments using anti-DVMAT-B_1_ heads were dissected and fixed for 1.5 hour at 23°C in 4% PFA, 0.1 M Na_2_HPO_4_/NaH_2_PO_4_ buffer, pH 7.4. Fixed brains were washed in PBS, 0.2% Triton X-100, incubated for 30 minutes in PBS, 0.2% Triton X-100, 5% FBS (block) and in 1∶100 rabbit anti-histamine or 1∶50 rat anti-DVMAT-B_1_ antibody overnight at 4°C followed by the corresponding secondary antibodies (1∶1000 of anti-rabbit Alexa Fluor 555, anti-rat Alexa Fluor 555, Molecular Probes, Eugene, OR, USA) for 4 hours at 23°C before mounting in Aqua PolyMount (Polysciences, Warrington, PA, USA).

To immunolabel cryostat sections using anti-N, heads were fixed in 4% PFA and embedded in OCT freezing solution (Sakura Finetechnical Co., Tokyo, Japan), frozen in liquid nitrogen and sections cut in a frontal plane at 10 µm thickness on a cryostat (Reichert-Jung 2800, Frigocut). The sections were processed for double-immunolabeling with two primary antibodies, mouse anti-Repo used at a dilution of 1∶10 and rabbit anti-DVMAT N-terminal at 1∶250. The corresponding secondary antibodies were used: Alexa-488 goat anti-mouse (Molecular Probes) at 1∶100 and Cy3 conjugated goat anti-rabbit (Jackson ImmunoResearch, West Grove, PA) at 1∶400. Labeled sections were mounted in Vectashield beneath no. 0 cover glasses.

The anti-DVMAT-B_2_ antiserum (1∶100), an affinity-purified rabbit antiserum ap63 against Tan peptides (1∶1000) [37], and a rabbit anti-Ebony antiserum against a 221-aa peptide spanning amino acids Gly438 to Asp658 of the Ebony protein [45] (1∶750) were employed for additional co-localization experiments. Primary antisera were applied overnight at 4°C. Secondary Cy2-, Cy3- or Cy5- conjugated antibodies (Dianova, Hamburg, Germany) were incubated either for 2 hours at 23°C or overnight at 4°C at a dilution of 1∶200 to 1∶1000. Slices were mounted in DakoCytomation Glycergel (DakoCytomation, Hamburg, Germany). For double-labeling with anti-histamine and anti-DVMAT-B_2_, a modified fixation procedure was applied: flies were fixed in 4% 2-ethyl-3-(3-dimethylaminopropyl) carbodiimide (EDAC) in 0.1 M phosphate buffer, pH 7.4, for 2 hours followed by 4% paraformaldehyde for 3 hours at 4°C. Fly heads were embedded in Tissue-Tec (Sakura Finetec, Zoeterwude, Netherlands), frozen in liquid nitrogen and sectioned at 10 µm. Histamine was detected with a rabbit anti-histamine antiserum (ImmunoStar, Hudson, WI, USA) at a 1∶500 dilution. Primary antisera were applied overnight at 4°C. Secondary antibodies were incubated for 2 hours at 23°C.

### EM Immunocytochemistry

#### Pre-embedding method

Heads were fixed in 2% PFA and 3.75% acrolein in 0.1 M PB, embedded in 7% agarose and 80 µm slices cut in a horizontal plane using a Vibratome. The sections were processed for immunohistochemistry with the rabbit polyclonal anti-N-terminal epitope of DVMAT (anti-N) used at a dilution of 1∶25. A goat anti-rabbit 1.4 nm gold-conjugated secondary antibody (Nanoprobes, Yaphank, NY, USA; Cat. no. 2003) was used at 1∶100. Labeled slices were postfixed with 2% glutaraldehyde, silver enhanced using an Amersham kit (Cat. no. RPN491; Amersham, Bucks, UK), then postfixed with 0.5% osmium tetroxide. After dehydration and embedding in PolyBed 810, 70 nm ultrathin sections were contrasted with uranyl acetate and Reynold's lead citrate and examined using an FEI Tecnai 12 electron microscope operated at 80 kV, and images collected with a Kodak Megaview II digital camera using AnalySIS software (SIS GmbH; Münster, Germany).

### High-Performance Liquid Chromatography (HPLC)

The histamine content of adult heads was analyzed by HPLC with fluorometric detection. Heads were homogenized and lysates prepared as described [40]. Histamine in the head lysates was derivatized with o-phthalaldehyde (OPA) before automated injection onto the HPLC column. The OPA derivatizing agent was prepared by adding 40 microliters of OPA (100 mg/ml of ethanol) and 5 microliters of beta-mercaptoethanol to 3.95 ml of 0.125 M boric acid buffer, pH 10. A 30-microliter aliquot of OPA reagent was reacted with the sample for 30 s. The OPA-amino acid adducts were resolved on a reverse-phase 3×150 mm column (Betabasic-18, 3 microns, C18, Thermo Electron) with sodium acetate (35 mM, pH 5.9 adjusted with glacial acetic acid), 1% tetrahydrofuran, as aqueous solvent. The organic mobile phase consisted of 70% acetonitrile, 15% methanol, and 15% sodium acetate (35 mM final concentration), pH 7.65 (adjusted with glacial acetic acid). The flow rate was 0.6 ml/min with a gradient profile as follows: 17–32% in 12 min, 32–40% in 3.5 min. The column was washed with a gradient of 40–100% in 0.5 min, held at 100% for 1 min, and returned to 17% in 0.5 min. The column was equilibrated at 17% for 6 min before injection of the next sample. Complete analysis required 24 min. The limit of detection was 5 fmol. This whole procedure, including data collection and calculations, was automated using Gilson hardware and software.
